# Can ^13^C stable isotope analysis uncover essential amino acid provisioning by termite-associated gut microbes?

**DOI:** 10.7717/peerj.1218

**Published:** 2015-08-27

**Authors:** Paul A. Ayayee, Susan C. Jones, Zakee L. Sabree

**Affiliations:** 1 Department of Evolution, Ecology and Organismal Biology, The Ohio State University, Columbus, OH, USA; 2 Department of Entomology, The Ohio State University, Columbus, OH, USA

**Keywords:** Essential amino acid, Gut microbiome, *Reticulitermes flavipes*, ^13^C-stable isotope analysis, Symbiosis

## Abstract

Gut-associated microbes of insects are postulated to provide a variety of nutritional functions including provisioning essential amino acids (EAAs). Demonstrations of EAA provisioning in insect-gut microbial systems, nonetheless, are scant. In this study, we investigated whether the eastern subterranean termite *Reticulitermes flavipes* sourced EAAs from its gut-associated microbiota. *δ*^13^C_EAA_ data from termite carcass, termite gut filtrate and dietary (wood) samples were determined following ^13^C stable isotope analysis. Termite carcass samples (−27.0 ± 0.4‰, mean ± s.e.) were significantly different from termite gut filtrate samples (−27.53 ± 0.5‰), but not the wood diet (−26.0 ± 0.5‰) (*F*_(2,64)_ = 6, *P* < 0.0052). *δ*^13^C_EAA_-offsets between termite samples and diet suggested possible non-dietary EAA input. Predictive modeling identified gut-associated bacteria and fungi, respectively as potential major and minor sources of EAAs in both termite carcass and gut filtrate samples, based on *δ*^13^C_EAA_ data of four and three EAAs from representative bacteria, fungi and plant data. The wood diet, however, was classified as fungal rather than plant in origin by the model. This is attributed to fungal infestation of the wood diet in the termite colony. This lowers the confidence with which gut microbes (bacteria and fungi) can be attributed with being the source of EAA input to the termite host. Despite this limitation, this study provides tentative data in support of hypothesized EAA provisioning by gut microbes, and also a baseline/framework upon which further work can be carried out to definitively verify this function.

## Introduction

Associations between termites and their gut microbes are some of the most well-studied symbioses. The majority of lower termites (all families except Termitidae) are wood feeders that thrive on these nitrogen-limited diets (Mattson, 1980) by relying upon gut microbes that can fix atmospheric nitrogen (Lilburn et al., 2001; Meuti, Jones & Curtis, 2010). Termites are incapable of meeting nutritional demands for nitrogen-rich metabolites such as proteins, in the absence of these microbes. Termites also rely on gut microbes to metabolize plant tissues, comprised largely of cellulose, into assimilable carbon. Digestion of cellulose and hemicellulose is attributed to a consortium of termite, bacteria and protist-derived cellulases that ultimately liberate carbon in plant tissues (Scharf et al., 2011; Tartar et al., 2009; Warnecke et al., 2007). Evidence of ^13^C-metabolite transfer between protists and associated gut bacteria in the desert damp wood termite, *Paraneotermes simplicicornis,* further confirms the flow of nutrients in the termite gut following ^13^C-cellulose degradation by associated protists (Carpenter et al., 2013). An additional microbe-specific function that benefits the termite host is acetogenesis/carbon dioxide fixation (Breznak & Kane, 1990; Pester & Brune, 2006). Together, nitrogen fixation, and acetogenesis provide ammonia and acetate, respectively, which the host can use for biosynthetic and metabolic processes. Oxygen scavenging and removal of excess hydrogen via methanogenesis are additional microbe-specific functions that are essential to maintaining the physiological and biochemical conditions within the gut microenvironment, ensuring that the aforementioned processes can continue (Brune & Friedrich, 2000).

An important aspect of wood-feeding insects’ nutritional ecology is the acquisition of essential amino acid (EAAs) because these cannot be generated by the host *de novo* (Douglas, 2013). Proctodeal trophallaxis (mouth-anus transfer of gut contents among nestmates), an essential colony feature, is thought to serve as one of the means by which termites acquire EAAs (Nalepa, Bignell & Bandi, 2001). Briefly, partially digested and undigested materials (with dead and living microbial fractions) are ingested from the anus of colony members and are used for inoculation or digestion (Kitade, 2004). Inoculation is thought to be more relevant for newly eclosed (hatched) and molted (inter-stadial growth) colony members, and digestion the norm in workers (Kitade, 2004). Similarly, notably higher normalized intensities of ^13^C-labelled EAAs detected in the lumen fluids of the midgut relative to the foregut and hindgut at 24 h, following feeding on ^13^C-cellulose in the damp wood termites (*Hodotermopsis sjostedti*) is attributed to proctodeal trophallaxis and subsequent digestion of microbial fractions (Tokuda et al., 2014). It still remains to be determined conclusively, that termites acquire and assimilate EAAs from gut microbes, since only the gut lumen fluid, but not actual termite tissue were sampled.

In this study, we investigated the acquisition of EAAs from associated gut microbiota of the eastern subterranean termite, *Reticulitermes flavipes,* using naturally occurring variations in the ^13^C/^12^C ratios of EAAs from bacteria, fungi, and plants. The premise of this approach is two-fold. First, because insects are incapable of *de novo* EAA biosynthesis and rely solely on dietary sources, the ^13^C-signature (determined via isotope ratio mass spectrometry) of an EAA in an insect consumer (*δ*^13^C_Consumer EAA_) is expected to approximate that of the diet (*δ*^13^C_Dietary EAA_), with little change in the ratio of ^13^C/^12^C stable isotopes in the carbon skeleton of that particular EAA (Caut, Angulo & Courchamp, 2008; McMahon et al., 2010; Newsome et al., 2011). The isotopic difference between consumer *δ*^13^C_EAA_ and dietary *δ*^13^C_EAA_(given by the delta notation; Δ*δ*^13^C = *δ*^13^C_Consumer EAA_ − *δ*^13^C_Dietary EAA_) is estimated to within 1‰ of the dietary *δ*^13^C_EAA_. Any significant deviation from the expected Δ*δ*^13^C of 1 suggests the possibility of alternate or additional sources of EAAs (Newsome et al., 2011; Tieszen et al., 1983).

The second premise relies on the fact that plants, bacteria and fungi are the only organisms capable of synthesizing EAAs and non-essential amino acids *de novo*. Additionally, bacteria, fungi, and plants have unique and distinct EAA signatures as a result of different biosynthetic pathways and processes that eventually lead to different ^13^C/^12^C stable isotopes ratios. The distinct *δ*^13^C_EAA_ signatures across these groups have been empirically demonstrated (Larsen et al., 2009) and used in several ecological studies (Larsen et al., 2011; Larsen et al., 2013; Vokhshoori, McCarthy & Larsen, 2014).

Thus, according to the first premise, a determined discrimination/offset factor (Δ*δ*^13^C) greater than 1 between the *δ*^13^C_EAA_ of a consumer and its diet, suggests the possibility of an additional/alternate source of EAAs for the consumer. The second premise enables the identification of the possible contributing source, within a predictive model framework, based on the unique *δ*^13^C_EAA_ signatures of plants, bacteria, and fungi. It is important to stress that, the ^13^C-offset determination serves only to determine the use of non-dietary EAAs if any, and not to identify biosynthetic origin of such non-dietary EAAs.

In this study, we investigated the ^13^C-offset between termites and their dietary substrates and assessed biosynthetic contributions from plant, bacteria and fungi to termite *δ*^13^C_EAA_ signature. Actual insect tissues in addition to gut lumen fluids were examined in order to conclusively determine the incorporation of microbial EAAs into the termite body.

## Materials and Methods

### Insects

*R. flavipes* originated from Orient, OH (39°46′19.99″N, 83°09′22.30″W) and were maintained in a laboratory colony in a plastic container fitted with a lid and provisioned with wood (a mixture of wood mulch and pine (*Pinus* spp.)), that was moistened periodically with distilled water. These colonies were maintained in the laboratory of Dr. Susan Jones. The termite colony originated from a single inbred colony that had been established during May 2010 by pairing a single male and female de-alate, and was maintained at room temperature (∼22 °C ± 2 °C) in the dark, under ambient laboratory conditions.

### Sample collection and preparation

After 8 weeks of feeding, 50 individual workers were removed from the colony and surface sterilized by rinsing once in 10x Coverage Plus (Steris, Mentor, Ohio, USA) and twice in sterile distilled water. A total of 5 termite samples (*n* = 5, each made up of 10 pooled workers) were obtained. The entire alimentary system was removed from each worker and placed in a 1x phosphate buffered saline solution (PBS). The remaining termite carcass was place in a 1.5 ml Eppendorf tube (Eppendorf, Hauppauge, New York, USA). Hence, each termite sample was subdivided into the termite carcass (*n* = 5) and its gut fraction (*n* = 5). Pooled termite guts were homogenized in PBS and filtered through a 0.45 µm membrane filter (EMD Millipore, Billerica, Massachusetts, USA) to eliminate insect debris. Gut filtrates were stored at −80 °C for 48 h prior to lyophilization. Wood samples (*n* = 4) from the termite colony were also ground into a coarse powder in a coffee mill and frozen at −80 °C for 48 h before lyophilization. Termite and wood samples then were sent to the Stable Isotope Facility (SIF) at UC Davis, Davis, California, USA, for ^13^C-stable isotope analysis.

### EAA stable isotope analysis

Freeze-dried termite and wood samples were acid hydrolyzed and derivatized resulting in the addition of a known carbon residue to the analytes of interest (Walsh, He & Yarnes, 2014). Non-analyte carbon correction was subsequently performed to correct for the addition of carbon during the derivatization process (Doherty, Jones & Evershed, 2001; Walsh, He & Yarnes, 2014). Approximately 0.2–0.5 µl aliquots of derivatized samples were injected into a splitless liner at 250 °C with a helium flow rate of 2.8 mL/min. Compound-specific isotope ^13^C-amino acid analysis (CSI- ^13^C_AA_) was performed using the TRACE GC Ultra gas chromatograph (GC; Thermo Fisher Scientific, Waltham, Massachusetts, USA) coupled to a Delta V Advantage isotope ratio mass spectrometer via the GC Combustion Interface III (Thermo Electron, Bremen, Germany) using the high polarity VF-23ms capillary column (Agilent Technologies, Santa Clara, California, USA). Combustion and reduction furnace temperatures were 950 °C and 650 °C, respectively. *δ*^13^C isotopic abundances are reported as *δ*^13^C values relative to the standard Vienna Pee Dee Belemnite (V-PDB) scale. *δ*^13^C_EAA_ quantified from termite carcasses, termite gut filtrates, and wood samples, following a non-analyte correction relative to internal amino acid standards, and used in the statistical analyses.

### Statistical analyses

Mixed model analysis and mean separations (Student’s *t*-test) were carried out on *δ*^13^C_EAA_ data using JMP 10 (SAS Inc., North Carolina, USA). Overall ^13^C-offset between termite (*δ*^13^C_Termite_) and wood (*δ*^13^C_Wood_) samples was determined as Δ*δ*^13^C = (*δ*^13^C_Termite_–*δ*^13^C_Wood_). Individual patterns of ^13^C-offset across EAAs between termite and wood samples was determined as Δ*δ*^13^C_EAA_ = (*δ*^13^C_Termite EAA_–*δ*^13^C_Wood EAA_).

### Calibration and model validation

An inter-lab calibration was performed to minimize instrumental error and/or variability between *δ*^13^C_EAA_ data from our study and from Larsen et al. (2013) for representative fungi (*n* = 9), bacteria (*n* = 11), and plants (*n* = 12). The predictive model was validated using the reference bacteria, fungi and plant samples as classifiers to ascertain distinctness of each group. Additionally, *δ*^13^C_EAA_ data obtained from the fungus *Fusarium solani* (*n* = 2), used in a previous study and analyzed at the same facility as these samples were, was used to further validate the separation of the classifiers by the model based on the particular EAAs used in the study.

This was followed by a supervised discriminant analysis to determine group membership of termite samples (carcass and gut filtrate) and wood samples to the respective classifier groups (Larsen et al., 2013). Classification of the training data and samples was performed using the jackknifed predictions. Linear discriminant function analysis (LDA) was carried out using the R package MASS (Venables & Ripley, 2002). We considered wood samples as predictors in the predictive modeling and not as classifiers.

### Ethics statement

No animal rights were violated in the execution of this study and conditions were within the guidelines of the Ohio State University’s Office of Responsible Research Practices.

## Results

The *δ*^13^C of all EAAs were quantified, and only those passing quality checking (having distinct and non-overlapping peaks obtained from the GC capillary column), were selected for further analysis. *δ*^13^C_EAA_ data were obtained for isoleucine, leucine, valine, phenylalanine, lysine and threonine from all samples (Table 1). Complete *δ*^13^C_EAA_ data, however, was available only for isoleucine, valine, phenylalanine and lysine across all samples. Leucine *δ*^13^C data was unavailable for three out of the five termite gut filtrate samples, due to failure to pass quality control (absence of distinct and non-overlapping peaks obtained from the GC capillary column). Similarly, Threonine *δ*^13^C data was unavailable for four out of five termite gut filtrate samples. Threonine was excluded from all further analyses. Leucine, however, was used in calculating the ^13^C-offset between termite samples and the wood diet, because there were two termite gut filtrate samples, for which this could be calculated relative to wood; but was omitted from the predictive model analysis due to the missing data points (Table S1).

**Table 1:** Summary ^13^C-data for all samples. Mean *δ*^13^C_EAA_ (mean of two technical replicates) of termite and wood samples following ^13^C-analysis at the UC Davis Stable Isotope Facility, showing data for all EAAs measured.

Samples	Ile	Leu	Lys	Phe	Thr	Val
Termite carcass 1	−26.59	−29.69	−19.70	−26.84	−16.82	−27.40
Termite carcass 2	−26.56	−29.85	−19.28	−26.54	−18.90	−26.79
Termite carcass 3	−27.62	−31.20	−21.72	−27.25	−17.98	−28.92
Termite carcass 4	−27.97	−31.51	−24.37	−27.66	−16.46	−28.77
Termite carcass 5	−26.11	−29.72	−20.04	−26.25	−14.65	−26.89
Termite gut filtrate 1	−42.35	N/A	−18.93	−25.37	N/A	−27.08
Termite gut filtrate 2	−29.26	N/A	−20.80	−28.16	N/A	−27.68
Termite gut filtrate 3	−31.30	−35.33	−18.05	−27.47	N/A	−28.79
Termite gut filtrate 4	−32.96	N/A	−19.78	−27.40	N/A	−27.05
Termite gut filtrate 5	−28.49	−32.16	−21.98	−27.92	−20.03	−27.41
Wood 1A	−21.54	−26.27	−19.49	−26.35	−18.96	−24.83
Wood 1B	−27.45	−30.26	−24.46	−29.78	−24.67	−29.53
Wood 2B	−23.78	−29.66	−20.36	−27.44	−16.95	−27.74

**Notes.**

N/A not available

### *δ*^13^C_EAA_ analysis summary and ^13^C-offset (Δ*δ*^13^C_EAA_) between termite samples and wood diet

The overall model was significant (*F*_(14,52)_ = 13.7, *P* < 0.0001, R-square = 0.80), with significant group (*F*_(2,64)_ = 6.0, *P* < 0.0052) and amino acid (*F*_(4,62)_ = 35, *P* < 0.0) effects, as well as significant group*amino acid interaction (*F*_(8,58)_ = 5.1, *P* < 0.0001). Termite gut filtrate (−27.53 ± 0.5‰) (mean ± s.e) was significantly different from the wood diet (−26 ± 0.5‰) and the termite carcass (−27.0 ± 0.4‰)(Table 1A). Termite carcass and wood diet were not significantly different from each other. Termite carcass and termite gut filtrate samples were respectively −1.0 ± 0.4‰ and −2.3 ± 0.4‰ ^13^C-depleted relative to wood the diet (Table 2A).

**Table 2:** Summary statistics of between sample comparisons. (A) Mean *δ*^13^C_EAA_ and Δ*δ*^13^C-offsets between termite samples (carcass and gut filtrate) and wood diet. Shown are mean values for termite (*n* = 5) and wood (*n* = 4) samples. Different letters represent a significant difference between groups (student’s *t*-test) (*F*_(2,63)_ = 6.0, *P* < 0.004). (B) Group*amino acid interaction mean *δ*^13^C_EAA_ values for each amino acids across all samples (*F*_(8,58)_ = 5.1, *P* < 0.0001) (student’s *t*-test).

(A) Sample	*δ*^13^C data (Mean ± s.e ‰)	Δ*δ*^13^C
Termite carcass	−27.0 ± 0.4 (B)	−1.0
Termite gut filtrate	−28.3 ± 0.5 (A)	−2.3
Wood	−26.0 ± 0.5 (B)	0
(*F*_(2,64)_ = 6.0, *P* < 0.0052)		

(B) Amino acid*group	*δ*^13^C data (Mean ± s.e ‰)
***Ile, Termite gut filtrate***	***−32.87 ± 1 (E)***
***Ile, Termite carcass***	***−26.97 ± 1 (C)***
***Ile, Wood***	***−23.69 ± 1.1(B)***
Leu, Termite gut filtrate	−33.76 ± 1 (E)
Leu, Termite carcass	−30.39 ± 1 (D, E)
Leu, Wood	−29.03 ± 1.1 (D)
Lys, Termite gut filtrate	−19.90 ± 1 (A)
Lys, Termite carcass	−21.02 ± 1 (A, B)
Lys, Wood	−22.22 ± 1.1 (A, B)
Phe, Termite gut filtrate	−27.26 ± 1 (C)
Phe, Termite carcass	−26.91 ± 1 (C)
Phe, Wood	−27.63 ± 1.1 (C, D)
Val, Termite gut filtrate	−27.60 ± 1 (C)
Val, Termite carcass	−27.75 ± 1 (D)
Val, Wood	−27.40 ± 1.1 (C)
(*F*_(8,58)_ = 5.1, *P* < 0.0001)	

**Figure 1:**
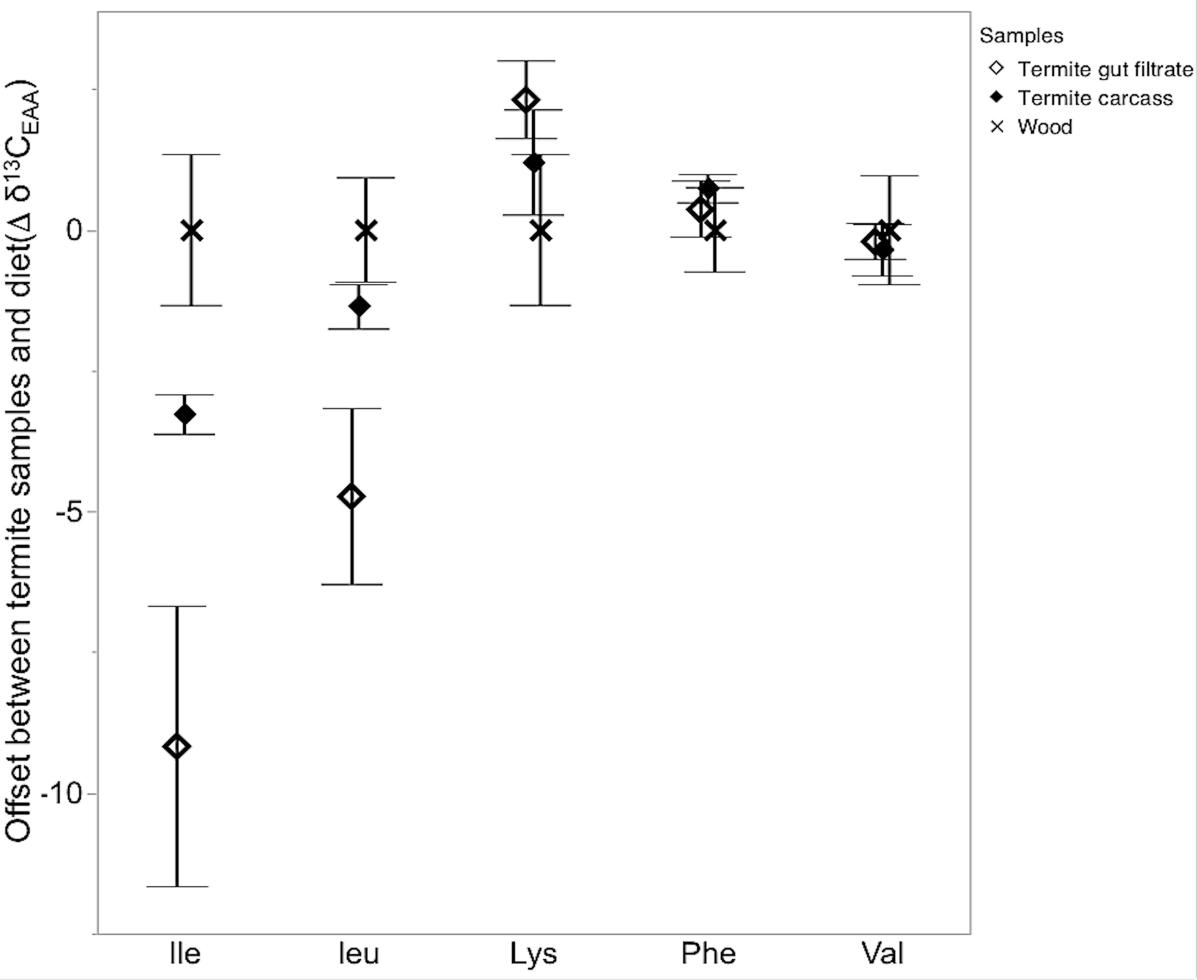
*δ*^13^C-offset between termite samples and wood diet. *δ*^13^C-offset (Δ*δ*^13^C_EAA_) (enrichment or depletion) of 5 five essential amino acids (EAAs) in termite carcass (termites) and termite gut filtrate samples relative to the wood diet EAAs; Δ*δ*^13^C_EAA_(‰) = (*δ*^13^C_Termite_EAA__–*δ*^13^C_Wood_ EAA). (*F*_(2,63)_ = 6.2, *P* < 0.004). Shown are mean values for 5 replicates per termite sample (termite and termite gut filtrate) and 4 replicates for wood diet. The EAAs were isoleucine (Ile), lysine (Lys), phenylalanine (Phe), and valine (Val). Error bars represent standard errors of the mean.

The pattern of ^13^C-offset of the five-measured EAAs in the termite samples relative to the wood diet is presented in Fig. 1. The ^13^C-offsets presented in Fig. 1 are not intended to resolve the biosynthetic origins of EAAs, merely to determine if the calculated Δ*δ*^13^C_EAA_ between termite samples and wood provides an indication of the use of non-dietary EAAs (i.e., equal to or greater than 1‰). Briefly, phenylalanine, lysine and valine from the termite samples (carcass and gut filtrate) and wood diet were not significantly different from the each other; even though lysine in the termite samples was ^13^C-enriched relative to the lysine in the wood-diet, and phenylalanine and valine in termite samples were ^13^C-depleted, relative to the wood diet (Fig. 1) (Table 2B). Isoleucine and leucine from termite gut filtrate samples was significantly ^13^C-depleted relative to termite carcass and wood diet. Only isoleucine in termite carcass samples was significantly ^13^C-depleted relative to the wood diet (Fig. 1) (Table 2B).

### Validation of predictive model and classification of termite sample EAAs

In the linear discriminant analysis (LDA) plots, the 95% confidence limits decision regions for each group/classifier are depicted as ellipses around the classifiers and the decision boundaries between the groups/classifiers as lines. After establishing the discrimination model, we then predicted posterior probabilities, i.e., the probability that a particular sample belonged to one or another of the three groups. The greater the distance of a particular consumer from the centroid of a classification group, i.e., potential EAA source, the greater the probability mixing of EAA sources occurred. Given the distinct discrimination scores between the classification groups, we interpreted discriminant scores of termite samples falling outside the 95% confidence limits of the food source, wood (plants) as strong indications of symbiotic EAA provisioning.

**Figure 2:**
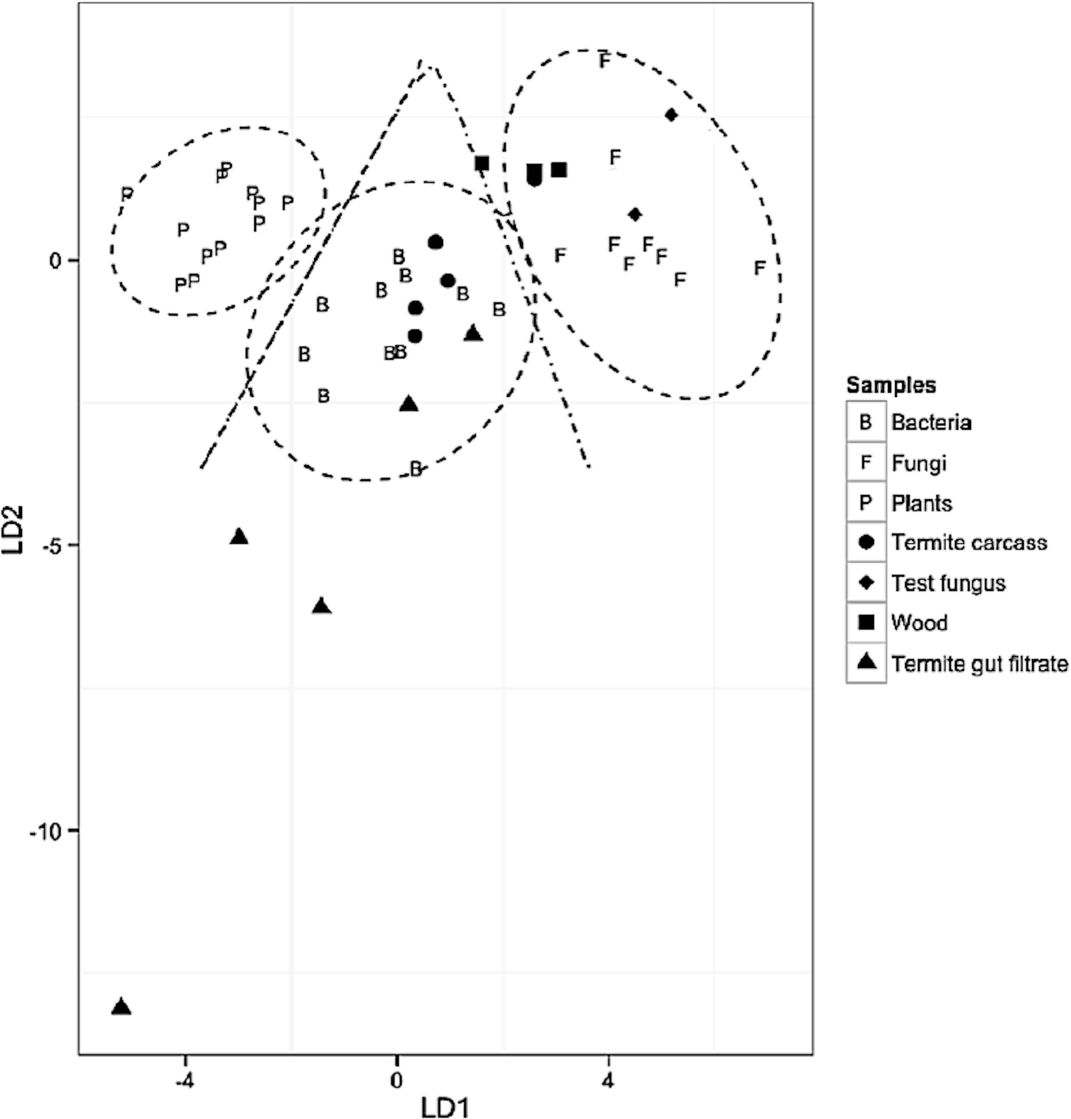
First discriminant analysis of termite, bacteria, fungi and plant samples. Predictive modeling (LDA) using *δ*^13^C_EAA_ data based on three classifier groups (plants (*n* = 12), fungi (*n* = 9), and bacteria (*n* = 11)) and three predictor groups (termite carcass (*n* = 5), termite gut filtrate (*n* = 5), and wood diet (*n* = 3)) using the EAAs; **isoleucine** (Ile), **lysine** (Lys), **phenylalanine** (Phe), and **valine** (Val). Wilks’ lambda = 0.09, *P* < 0.0001; LD1 = 92.6%, LD2 = 7.4%. 95% confidence limits decision regions for each group/classifier are depicted as ellipses around the classifiers and the decision boundaries between the groups/classifiers as lines.

The predictive model was validated, based on the correct classification of bacteria (*n* = 11), fungi (*n* = 9) and plants (*n* = 12) to their respective groups (*F*_(8,54)_ = 25, *P* < 0.0001; Wilk’s lambda = 0.04, a test of appropriateness of classifiers in predicting group membership of predictors), using the *δ*^13^C_EAA_ values of the EAAs, isoleucine, phenylalanine, valine and lysine (Fig. 2). Additionally, two test fungus (*Fusarium solani*) samples were correctly classified as fungi. The classification of these fungal samples further validated the appropriateness of the model using the selected EAAs (Fig. 2). Wood was not used in the training data (Table S1), because we were interested in determining whether it would be correctly classified with the plant classifier. The posterior probabilities associated with the model classifications are summarized for both the model classifiers and the termite and wood samples in Table S2.

Two wood samples fell within the 95% confidence limit decision region of the fungal classifier. This is suggestive of possible fungal infestation of the wood materials. The remaining third wood sample was in-between the fungal and bacterial classifier decision boundaries. Four termite carcass samples and two termite gut filtrate samples had discriminant scores within the 95% confidence limit decision region of the bacteria classifier, suggestive of possible bacterial EAA input (Fig. 2). One termite carcass sample was within the 95% confidence limit decision region of the fungal classifier, suggestive of fungal EAA input in that sample (Fig. 2). Three termite gut filtrate samples were outside of the 95% confidence limit decision region of the bacterial classifier, but were within the decision boundary of the bacterial classifier. This is taken to suggest likely bacterial EAA input in these samples (Fig. 2). The displacement of these termite gut filtrate samples is attributed to their ^13^C-depleted isoleucine values (Table S1).

**Figure 3:**
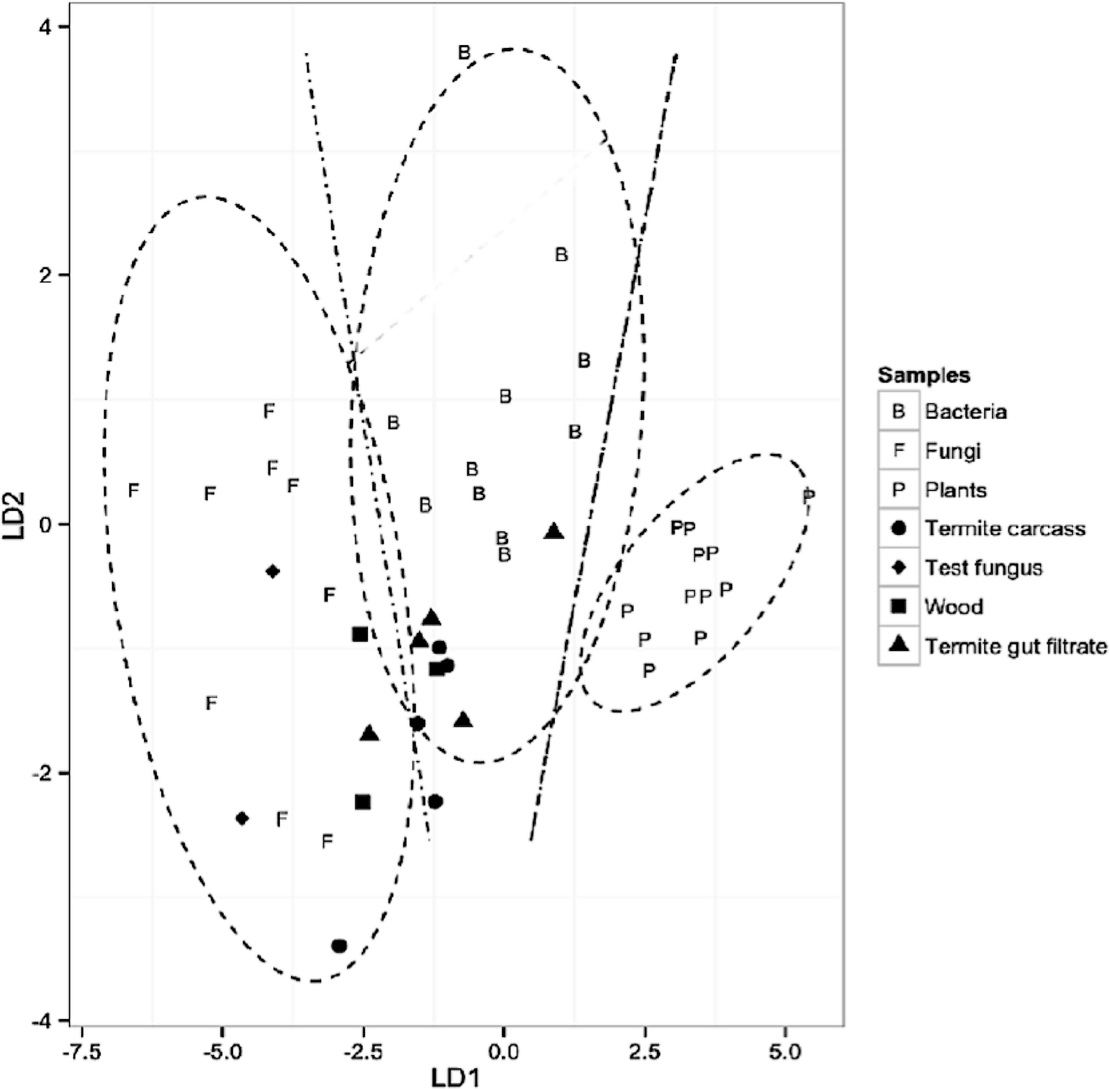
Second discriminant analysis of termite, bacteria, fungi and plant samples. Predictive modeling (LDA) using *δ*^13^C_EAA_ data based on three classifier groups (plants (*n* = 12), fungi (*n* = 9), and bacteria (*n* = 11)) and three predictor groups (termite carcass (*n* = 5), termite gut filtrate (*n* = 5), and wood diet (*n* = 3)) using the EAAs; **lysine** (Lys), **phenylalanine** (Phe), and **valine** (Val). Wilks’ lambda = 0.09, *P* < 0.0001; LD1 = 95.3%, LD2 = 4.6%. 95% confidence limits decision regions for each group/classifier are depicted as ellipses around the classifiers and the decision boundaries between the groups/classifiers as lines.

The performance of the classification model without isoleucine from all samples was investigated, due to concerns about the influence of isoleucine on the displacement of samples in the LDA plot (Fig. 1). Bacteria, fungi and plant samples were correctly classified into distinct groups (*F*_(8,54)_ = 28.9, *P* < 0.0001; Wilk’s lambda = 0.06, a test of appropriateness of classifiers in predicting group membership of predictors) (Table S3). As with the previous analysis, the test fungus, *F. solani* samples were similarly correctly classified as fungal in origin (Fig. 3), further validating the model and the classification in the absence of isoleucine. Omitting isoleucine from the model resulted in the placement of four termite gut filtrate samples within the 95% confidence limit decision region of the bacterial classifier, and the fifth one within the fungal classifier decision region (Fig. 3). Four termite carcass samples were classified as bacterial in origin, and three were located within the decision region of the bacterial classifier group. The fifth termite carcass sample was classified as fungal (Fig. 3). Classification of both termite carcass and termite gut filtrate samples in both models (Figs. 2 and 3) was essentially similar (Table S3). Omitting isoleucine in the second analysis, nonetheless, minimized the variance between samples and reduced the skewing of the samples within the LDA plot (Fig. 3). Based on the ^13^C-offset data and the results from both predictive model analyses, the hypothesis of gut microbial EAA is tentatively substantiated. Nonetheless, the sourcing of EAAs from extracellular wood-degrading fungi by termites in this study remains an additional/alternate possibility.

## Discussion

The membership of the gut microbiome of wood-feeding termites like *R. flavipes* is varied and members perform several key important functions related to the host’s nutritional ecology. Essential amino acid provisioning by these gut microbes have been proposed, but remain to be empirically determined. In this study, we sought to determine gut microbial EAA provisioning to termite hosts by taking advantage of the natural variations in the *δ*^13^C_EAA_ stable isotope signatures between plants, bacteria, and fungi.

A primary premise of this approach is that termite *δ*^13^C_EAA_ would closely resemble dietary *δ*^13^C_EAA_ (wood) in the absence of microbial provisioning. While we did not determine a significant difference between termite carcass and dietary *δ*^13^C_EAA_(Δ*δ*^13^C = 1) (Table 1), there were notable variations in the ^13^C-offset patterns of isoleucine, leucine, and lysine, between termites and the wood diet (Fig. 1). Most wood-feeding lower termites are notably ^13^C-depletd compared to fungus feeding and soil-feeding termites (Tayasu, 1998). Degradation of cellulose by host and microbe-derived cellulolytic processes liberates carbon from wood in the termite gut (Watanabe & Tokuda, 2010). However, there are multiple microbial processes taking place in the termite gut besides cellulose degradation, including reductive acetogenesis (Brune & Friedrich, 2000), which affects the ^13^C-signatures of carbon liberated due to cellulose degradation and the carbon finally incorporated into insect host tissues. The higher incidences of microbial acetogenesis relative to methanogenesis in wood-feeding lower termites, followed by absorption of the newly formed and ^13^C-depleted acetate, is proposed to be responsible for the determined negative ^13^C-discrimination between wood-feeding lower termites and their woody diets (Bignell et al., 1995; Tayasu, 1998). While the Δ*δ*^13^C_EAA_ between termite carcass and diet was not greater than the posited 1‰, *δ*^13^C-depletion has been previously reported between termite and their wood diet based on bulk *δ*^13^C data (Bignell et al., 1995; Tayasu, 1998). Thus for termites, based on the diverse composition and functionalities of the gut microbiota, perhaps a ^13^C-offset of 1‰ may be sufficient to indicate non-dietary EAA input. Additionally, the individual variations in the ^13^C-offsets across all EAAs between the wood diet and termite carcass and gut filtrate samples, possibly further suggests non-dietary EAA input despite the overall ^13^C-offset of 1‰ (Fig. 1).

An investigation of the biosynthetic origins of EAAs in termite carcass and gut filtrate samples using the predictive model classified a majority of termite samples as bacterial in origin (Figs. 2 and 3), and diet (wood) samples as mainly fungal. Analyzing the data, with and without isoleucine, demonstrated the suitability of the EAAs used to adequately separate fungi, bacteria and plants in the predictive models. Unfortunately, not all EAAs passed quality control, and were therefore not available for use in further analyses. Of the 6 EAAs, measured from samples in this study, only 4 (Fig. 2) and 3 (Fig. 3) were used in the LDA. This is attributed partially to the difficulties inherent in the successful derivatization and analytical processes associated with quantifying *δ*^13^C_EAA_ data. Increasing the number of EAAs used in the model, would essentially offer greater resolution as well as increased confidence in the subsequent model classifications.

Despite the limitation regarding the number and kinds of EAAs used, results from both predictive models show moderate support for the assertion of gut microbial sources of EAAs to the termite host (Fig. 2). These results however, are not definitive and require further validation. The major reasons for this are the absence of significant ^13^C-offsets between termite samples and three of the EAAs in the study (lysine, valine and phenylalanine), and the classification of diet (wood samples) as fungal in the predictive model (Fig. 2, Table S2). This suggests, perhaps, that the wood diet was compromised and was not appropriate for this effort. Fungal growth has been observed to quickly overtake laboratory termite colonies when populations are either small or stressed (S Jones, pers. comm., 2015). The classification of only one of the termite carcass samples as fungal is of importance, but does not detract from the bacterial origin of EAAs in the other four termite carcass samples (Figs. 2 and 3).

The relative significance/importance of fungal EAA input compared to bacterial EAA input was not the objective of this study. Fungal origins of EAA, however, cannot be ruled out, since fungi associated with termites may begin the cellulolytic process prior to termite ingestion, thus enhancing cellulose degradation (Hyodo et al., 2003). Ingested fungi may in turn be consumed and digested for EAAs, evidenced from the predominantly fungal EAA signature in one of the termite carcass samples. This scenario is not entirely unlikely, since fungal origins of EAA (as a component of gut microbial EAA input) have been previously documented in other insects such as, the solitary, wood-feeding *Anoplophora glabripennis* (Ayayee et al., in press).

Based on the social structure of *R. flavipes* colonies, and proctodeal trophallaxis between colony mates, (Nalepa, Bignell & Bandi, 2001; Shimada et al., 2013), digestion and assimilation of microbial EAA following proctodeal transfers is most likely the route for the bacterial/microbial EAA input observed in this study. Alternatively, direct absorption of microbial of EAA from across the walls of the hindgut paunch in termites is likely, in a manner similar to the uptake of acetate produced by hindgut microbial residents (Breznak & Kane, 1990).

Protists associated with termites are known to aid synergistically in cellulose degradation (Scharf et al., 2011), and serve as hosts to both ecto- and endo- symbiotic bacteria within the gut lumen (Ohkuma, 2008). Little is known about the essential amino acid biosynthetic capabilities of protists in general, but it is likely, that being eukaryotes, they lack the machinery necessary for *de novo* EAA biosynthesis (Ginger et al., 2010), but are capable of utilizing EAAs as substrate for metabolism. It remains to be conclusively determined that termite gut-associated protists are incapable of EAA biosynthesis, and thus the possibility that termites may be acquiring protist-derived EAAs cannot be entirely ruled out. Termite gut-associated protists however, are known to be obligate hosts to a variety of ecto- and endo-symbiotic bacteria present in termite microbiomes (Ohkuma, 2008). The functions of these protist-associated bacteria include nitrogen fixation, reductive acetogenesis and methanogenesis, as well as limited cellulose degradation (Ohkuma, 2008). These associated bacteria are currently regarded as essential sources of metabolites such as EAAs for their protist hosts. The protists together with associated bacteria then, subsequently serve as sources of EAA for the termite hosts upon digestion.

Overall, this study demonstrates the applicability of the ^13^C-fingerprint approach in investigating biosynthetic origins of EAA in insect-microbe systems. Additional improvements to the experimental design (careful selection of experimental dietary materials), increased sample numbers, and optimized procedures/protocols for *δ*^13^C_EAA_ data generation via isotope ratio mass spectrometry, are needed, in order for definitive establishment of the role of gut-associated microbes of termites as sources of EAA.

## Conclusions

In summation, this study provides promising evidence in support of putative gut microbial (bacterial and fungal) EAA provisioning in termites. Though the results presented herein are not exhaustive, they serve as the baseline for further work, investigating microbial EAA provisioning functions in greater detail. Finally, the results presented provide a framework/approach to, investigating gut microbial EAA provisioning in similar, and other insect-microbe symbiotic system using *δ*^13^C stable isotope analysis.

## Supplemental Information

10.7717/peerj.1218/supp-1 Table S1 Summary raw calibrated ^13^C data Sample source and all mean inter-lab calibrated essential amino acid values (*δ*^13^C_EAA_) used in this study. Means are based on 2 technical replicates for each biological sample and 3 technical replicates for fungi, bacteria, and plant reference samples.

10.7717/peerj.1218/supp-2 Table S2 Validation of model and sample classifications for second discriminant analysis in Fig. 2
Posterior probabilities of the classifier samples (fungi, bacteria, and Plants) and experimental group samples used in the predictive model plot in Fig. 2. (Wilks’ lambda = 0.09, *P* < 0.0001).

10.7717/peerj.1218/supp-3 Table S3 Validation of model and sample classifications for second discriminant analysis in Fig. 3
