# Supplementary material for: Can 13C stable isotope analysis uncover essential amino acid provisioning by termite-associated gut microbes?
Source: PeerJ. 2015 Aug 27;3:e1218. doi: 10.7717/peerj.1218 (PMC4556154; doi:10.7717/peerj.1218)
Supplement: Table S1 — Sample source and all mean inter-lab calibrated essential amino acid values (δ13CEAA) used in this study. Means are based on 2 technical replicates for each biological sample and 3 technical replicates for fungi, bacteria, and plant reference samples. [file peerj-03-1218-s001.docx]

**Supplementary material: Table S1**. Sample source and all mean inter-lab calibrated essential amino acid values (δ^13^C_EAA_) used in this study. Means are based on 2 technical replicates for each biological sample and 3 technical replicates for fungi, bacteria, and plant reference samples (Larsen et al., 2013).

| **ID** | **Sample** | **Species** | **Category** | **LDA** | **Ile** | **Lys** | **Phe** | **Val** |
| --- | --- | --- | --- | --- | --- | --- | --- | --- |
| F1 | Fungi | *Ascomycota* | Fungi | Training set | -24 | -22.6 | -26.9 | -25.6 |
| F2 | Fungi | *Aureobasidium pullulans* | Fungi | Training set | -22.8 | -21 | -26.1 | -22.2 |
| F3 | Fungi | *Bionectria orhroleuca* | Fungi | Training set | -25.4 | -22.2 | -29.7 | -26.6 |
| F4 | Fungi | *Nectria vilior* | Fungi | Training set | -23.2 | -23.1 | -27.5 | -27.3 |
| F5 | Fungi | Unidentified | Fungi | Training set | -9.1 | -7.2 | -14.1 | -10.9 |
| F6 | Fungi | Unidentified | Fungi | Training set | -8.7 | -9 | -14.8 | -8.8 |
| F7 | Fungi | Unidentified | Fungi | Training set | -10 | -8.7 | -15 | -10.6 |
| F8 | Fungi | Mortierella alpi | Fungi | Training set | -6.6 | -5.5 | -12.9 | -9.2 |
| F9 | Fungi | Unidentified | Fungi | Training set | -9.8 | -7.9 | -14.8 | -12.1 |
| B1 | Bacteria | *Burkholderia xenovorans* | Bacteria | Training set | -12.5 | -4.9 | -18.3 | -14.1 |
| B10 | Bacteria | Bacteria G | Bacteria | Training set | -19.2 | -12.3 | -20.4 | -22 |
| B11 | Bacteria | Bacteria H | Bacteria | Training set | -19.1 | -13.1 | -21.5 | -22.7 |
| B12 | Bacteria | Bacteria J | Bacteria | Training set | -24.6 | -19.3 | -28.3 | -26.5 |
| B3 | Bacteria | *Klebsiella* sp. | Bacteria | Training set | -19.5 | -14.8 | -24.1 | -21.4 |
| B4 | Bacteria | *Rhodococcus* sp*.* | Bacteria | Training set | -24.3 | -15.7 | -28.5 | -26.9 |
| B5 | Bacteria | Bacteria B | Bacteria | Training set | -16.6 | -9.3 | -17.9 | -18.3 |
| B6 | Bacteria | Bacteria C | Bacteria | Training set | -17.6 | -9.8 | -19.7 | -21.6 |
| B7 | Bacteria | Bacteria D | Bacteria | Training set | -16.1 | -7.4 | -18 | -19.6 |
| B8 | Bacteria | Bacteria E | Bacteria | Training set | -16.6 | -9 | -17.3 | -18.2 |
| B9 | Bacteria | Bacteria F | Bacteria | Training set | -21.7 | -16 | -26.6 | -25.6 |
| T1 | Plants | *Quercus robur* | Plants | Training set | -29.7 | -20.1 | -32.1 | -37.7 |
| T10 | Plants | *Salix reticulata* | Plants | Training set | -25.7 | -17.7 | -26 | -31 |
| T11 | Plants | *Eriophorum angustifolium* | Plants | Training set | -23.8 | -16.3 | -26.5 | -30.5 |
| T12 | Plants | *Rumex arcticus* | Plants | Training set | -28.2 | -20.1 | -29.5 | -34.7 |
| T2 | Plants | *Alnus glutinosa* | Plants | Training set | -31.3 | -22.4 | -33.4 | -36.6 |
| T3 | Plants | *Salix* sp. | Plants | Training set | -24.8 | -17 | -26.6 | -31.9 |
| T4 | Plants | *Polygonum viviparum* | Plants | Training set | -27.9 | -18.4 | -28.4 | -33.8 |
| T5 | Plants | *Carex aquatilis* | Plants | Training set | -27.3 | -17 | -27.2 | -32.1 |
| T6 | Plants | *Calamagrostis cadensis* | Plants | Training set | -27.7 | -19.5 | -28.4 | -32.6 |
| T7 | Plants | *Menganthes trifoliata* | Plants | Training set | -28 | -18 | -28.2 | -32.7 |
| T8 | Plants | *Betula* | Plants | Training set | -25.7 | -18.4 | -27.4 | -31.1 |
| T9 | Plants | *Carex utriculata* | Plants | Training set | -28.3 | -18.8 | -28.5 | -33.2 |
| T1 | Termite | Termite carcass | Termite carcass | Termite carcass | -26.59 | -19.7 | -26.84 | -27.4 |
| T2 | Termite | Termite carcass | Termite carcass | Termite carcass | -26.56 | -19.28 | -26.54 | -26.79 |
| T3 | Termite | Termite carcass | Termite carcass | Termite carcass | -27.62 | -21.72 | -27.25 | -28.92 |
| T4 | Termite | Termite carcass | Termite carcass | Termite carcass | -27.97 | -24.37 | -27.66 | -28.77 |
| T5 | Termite | Termite carcass | Termite carcass | Termite carcass | -26.11 | -20.04 | -26.25 | -26.89 |
| TG1 | Termite gut | Termite gut filtrate | Termite gut filtrate | Termite gut filtrate | -42.35 | -18.93 | -25.37 | -27.08 |
| TG2 | Termite gut | Termite gut filtrate | Termite gut filtrate | Termite gut filtrate | -29.26 | -20.8 | -28.16 | -27.68 |
| TG3 | Termite gut | Termite gut filtrate | Termite gut filtrate | Termite gut filtrate | -31.3 | -18.05 | -27.47 | -28.79 |
| TG4 | Termite gut | Termite gut filtrate | Termite gut filtrate | Termite gut filtrate | -32.96 | -19.78 | -27.4 | -27.05 |
| TG5 | Termite gut | Termite gut filtrate | Termite gut filtrate | Termite gut filtrate | -28.49 | -21.98 | -27.92 | -27.41 |
| Wood 1A | Wood | Wood | Wood | Wood | -21.54 | -19.49 | -26.35 | -24.83 |
| Wood 1B | Wood | Wood | Wood | Wood | -27.45 | -24.46 | -29.78 | -29.53 |
| Wood 2B | Wood | Wood | Wood | Wood | -23.78 | -20.36 | -27.44 | -27.74 |
| ALB-FS-1 | *Fusarium sp.* | Test fungus | Test fungus | Predictor | -20.73 | -19.50 | -26.54 | -22.49 |
| ALB-FS-2-B | *Fusarium sp.* | Test fungus | Test fungus | Predictor | -23.19 | -23.44 | -27.54 | -25.29 |
