# Supplementary material for: Can 13C stable isotope analysis uncover essential amino acid provisioning by termite-associated gut microbes?
Source: PeerJ. 2015 Aug 27;3:e1218. doi: 10.7717/peerj.1218 (PMC4556154; doi:10.7717/peerj.1218)
Supplement: Table S2 — Posterior probabilities of the classifier samples (fungi, bacteria, and Plants) and experimental group samples used in the predictive model plot in Fig. 2. (Wilks’ lambda = 0.09, P < 0.0001). [file peerj-03-1218-s002.docx]

**Supplemental material: Table S2.** Posterior probabilities of the classifier samples (fungi, bacteria, and Plants) and experimental group samples used in the predictive model plot in Fig. 2. (Wilks’ lambda = 0.09, *P* < 0.0001).

| **Samples** | **Actual** | **Bacteria** | **Fungi** | **Plants** |
| --- | --- | --- | --- | --- |
| Fungi | Fungi | 0 | 100 | 0 |
| Fungi | Fungi | 0 | 100 | 0 |
| Fungi | Fungi | 0.9 | 99.1 | 0 |
| Fungi | Fungi | 0 | 100 | 0 |
| Fungi | Fungi | 0 | 100 | 0 |
| Fungi | Fungi | 0 | 100 | 0 |
| Fungi | Fungi | 0 | 100 | 0 |
| Fungi | Fungi | 0 | 100 | 0 |
| Fungi | Fungi | 0.01 | 99.9 | 0 |
| Bacteria | Bacteria | 100 | 0 | 0 |
| Bacteria | Bacteria | 99.4 | 0 | 0.6 |
| Bacteria | Bacteria | 99.4 | 0 | 0.6 |
| Bacteria | Bacteria | 99.5 | 0.5 | 0 |
| Bacteria | Bacteria | 93.2 | 6.8 | 0 |
| Bacteria | Bacteria | 99.4 | 0 | 0.6 |
| Bacteria | Bacteria | 100 | 0 | 0 |
| Bacteria | Bacteria | 88.4 | 0 | 11.6 |
| Bacteria | Bacteria | 93 | 0 | 7 |
| Bacteria | Bacteria | 100 | 0 | 0 |
| Bacteria | Bacteria | 99.8 | 0 | 0.2 |
| Plants | Plants | 0 | 0 | 100 |
| Plants | Plants | 0.5 | 0 | 99.5 |
| Plants | Plants | 0.3 | 0 | 99.7 |
| Plants | Plants | 0.02 | 0 | 99.98 |
| Plants | Plants | 0 | 0 | 100 |
| Plants | Plants | 0 | 0 | 100 |
| Plants | Plants | 0 | 0 | 100 |
| Plants | Plants | 0.06 | 0 | 99.94 |
| Plants | Plants | 0.96 | 0 | 99.04 |
| Plants | Plants | 0.12 | 0 | 99.88 |
| Plants | Plants | 2.7 | 0 | 97.3 |
| Plants | Plants | 0.11 | 0 | 99.89 |
| **Samples** | **Predicted** | **Bacteria** | **Fungi** | **Plants** |
| Termite carcass | Bacteria | 100 | 0 | 0 |
| Termite carcass | Bacteria | 100 | 0 | 0 |
| Termite carcass | Bacteria | 99.7 | 0.3 | 0 |
| Termite carcass | Fungi | 0.8 | 99.2 | 0 |
| Termite carcass | Bacteria | 99.8 | 0.2 | 0 |
| Termite gut filtrate | Bacteria | 100 | 0 | 0 |
| Termite gut filtrate | Bacteria | 100 | 0 | 0 |
| Termite gut filtrate | Bacteria | 99.2 | 0 | 0.8 |
| Termite gut filtrate | Bacteria | 100 | 0 | 0 |
| Termite gut filtrate | Bacteria | 99.7 | 0.3 | 0 |
| Wood | Fungi | 0.06 | 99.94 | 0 |
| Wood | Fungi | 0.5 | 95 | 0 |
| Wood | Fungi | 35 | 65 | 0 |
| *Fusarium solani* | Fungi | 0 | 100 | 0 |
| *Fusarium solani* | Fungi | 0 | 100 | 0 |
