# Supplementary material for: Can 13C stable isotope analysis uncover essential amino acid provisioning by termite-associated gut microbes?
Source: PeerJ. 2015 Aug 27;3:e1218. doi: 10.7717/peerj.1218 (PMC4556154; doi:10.7717/peerj.1218)
Supplement: Table S3 [file peerj-03-1218-s003.docx]

**Supplemental material; Table S3.** Posterior probabilities of the classifier samples (fungi, bacteria, and Plants) and experimental group samples used in the predictive model (without isoleucine) plot in Fig. 3. (Wilks’ lambda = 0.06, *P* < 0.0001).

| **Samples** | **Actual** | **Bacteria** | **Fungi** | **Plants** |
| --- | --- | --- | --- | --- |
| Fungi | Fungi | 0 | 100 | 0 |
| Fungi | Fungi | 0 | 100 | 0 |
| Fungi | Fungi | 0.85 | 99.15 | 0 |
| Fungi | Fungi | 0.04 | 99.96 | 0 |
| Fungi | Fungi | 0.05 | 99.95 | 0 |
| Fungi | Fungi | 0 | 100 | 0 |
| Fungi | Fungi | 0 | 100 | 0 |
| Fungi | Fungi | 0.08 | 99.92 | 0 |
| Fungi | Fungi | 0.2 | 99.8 | 0 |
| Bacteria | Bacteria | 100 | 0 | 0 |
| Bacteria | Bacteria | 99.3 | 0 | 0.7 |
| Bacteria | Bacteria | 99.5 | 0 | 0.5 |
| Bacteria | Bacteria | 97.1 | 2.9 | 0 |
| Bacteria | Bacteria | 88.9 | 11.1 | 0 |
| Bacteria | Bacteria | 99.2 | 0 | 0.8 |
| Bacteria | Bacteria | 99.94 | 0.06 | 0 |
| Bacteria | Bacteria | 88.2 | 0 | 11.8 |
| Bacteria | Bacteria | 90.7 | 0 | 9.3 |
| Bacteria | Bacteria | 99.88 | 0.05 | 0.07 |
| Bacteria | Bacteria | 99.88 | 0 | 0.12 |
| Plants | Plants | 0 | 0 | 100 |
| Plants | Plants | 0.5 | 0 | 99.5 |
| Plants | Plants | 0.4 | 0 | 99.6 |
| Plants | Plants | 0.03 | 0 | 99.97 |
| Plants | Plants | 0.2 | 0 | 99.8 |
| Plants | Plants | 0.04 | 0 | 99.96 |
| Plants | Plants | 0 | 0 | 100 |
| Plants | Plants | 0.04 | 0 | 99.96 |
| Plants | Plants | 0.9 | 0 | 99.1 |
| Plants | Plants | 0.08 | 0 | 99.92 |
| Plants | Plants | 3.5 | 0 | 96.5 |
| Plants | Plants | 0.09 | 0 | 99.91 |
| **Samples** | **Predicted** | **Bacteria** | **Fungi** | **Plants** |
| Termite carcass | Bacteria | 96.1 | 3.9 | 0 |
| Termite carcass | Bacteria | 94.4 | 5.6 | 0 |
| Termite carcass | Bacteria | 65.8 | 34.1 | 0.1 |
| Termite carcass | Fungi | 0.03 | 99.97 | 0 |
| Termite carcass | Bacteria | 57.1 | 43 | 0.012 |
| Termite gut filtrate | Bacteria | 97.3 | 2.32 | 0.34 |
| Termite gut filtrate | Bacteria | 80 | 20 | 0 |
| Termite gut filtrate | Bacteria | 89.8 | 0 | 10.24 |
| Termite gut filtrate | Bacteria | 92.6 | 7.4 | 0 |
| Termite gut filtrate | Bacteria | 3.01 | 96.99 | 0 |
| Wood | Fungi | 4.7 | 95.3 | 0 |
| Wood | Fungi | 0.8 | 99.2 | 0 |
| Wood | Fungi | 91.5 | 8.51 | 0 |
| *Fusarium solani* | Fungi | 0.015 | 99.98 | 0 |
| *Fusarium solani* | Fungi | 0 | 100 | 0 |
